# Supplementary material for: Transcriptomic Alterations Induced by Tetrahydrocannabinol in SIV/HIV Infection: A Systematic Review
Source: Int J Mol Sci. 2025 Mar 13;26(6):2598. doi: 10.3390/ijms26062598 (PMC11942185; doi:10.3390/ijms26062598)
Supplement: Supplementary file 1 [file ijms-26-02598-s001.zip › Table S1.pdf]

Supplementary Table S1

APA PsycInfo [Ovid], <1806 - 02/20/2025>

1. (HIV or HIV-1 or "human immunodeficiency virus" or AIDS or "acquired immunodeficiency syndrome").ti,ab.
2. (SIV or "simian immunodeficiency virus").ti,ab.
3. 1 or 2
4. (rhesus macaque or Macaca mulatta or rhesus).ti,ab.
5. (THC or "delta-9-tetrahydrocannabinol" or "Δ-9-tetrahydrocannabinol").ti,ab.
6. (gene expression or mRNA or microRNA or miRNA or transcriptome or epigenetic* or methylation or histone* or transcription or "RNA-seq" or "chip-seq" or immune marker* or "viral load").ti,ab.
7. 3 and 4 and 5 and 6

Embase [Ovid], <1974 - 02/20/2025>

1. (HIV or HIV-1 or "human immunodeficiency virus" or AIDS or "acquired immunodeficiency syndrome").ti.
2. exp HIV Infections/ or exp Acquired Immunodeficiency Syndrome/
3. (SIV or "simian immunodeficiency virus").ti.
4. exp Simian Immunodeficiency Virus/
5. 1 or 2 or 3 or 4
6. (rhesus macaque or Macaca mulatta or rhesus).ti,ab.
7. exp Macaca mulatta/
8. 6 or 7
9. 5 and 8
10. (THC or "delta-9-tetrahydrocannabinol" or "Δ-9-tetrahydrocannabinol").ti,ab.
11. exp Tetrahydrocannabinol/
12. 10 or 11
13. (gene expression or mRNA or microRNA or miRNA or transcriptome or epigenetic* or methylation or histone* or transcription or "RNA-seq" or "chip-seq").ti,ab.
14. (immune marker* or "viral load").ti,ab.
15. 13 or 14
16. 9 and 12 and 15

MEDLINE [Ovid], <1946 – 02/20/2025>

1. (HIV or HIV-1 or "human immunodeficiency virus" or AIDS or "acquired immunodeficiency syndrome").ti.
2. exp HIV Infections/ or exp Acquired Immunodeficiency Syndrome/
3. (SIV or "simian immunodeficiency virus").ti.
4. exp Simian Immunodeficiency Virus/
5. 1 or 2 or 3 or 4
6. (rhesus macaque or Macaca mulatta or rhesus).ti,ab.
7. exp Macaca mulatta/
8. 6 or 7
9. 5 and 8
10. (THC or "delta-9-tetrahydrocannabinol" or "Δ-9-tetrahydrocannabinol").ti,ab.
11. exp THC/
12. 10 or 11
13. (gene expression or mRNA or microRNA or miRNA or transcriptome or epigenetic* or methylation or histone* or transcription or "RNA-seq" or "chip-seq").ti,ab.
14. (immune marker* or "viral load").ti,ab.
15. 13 or 14
16. 9 and 12 and 15

PubMed [National Library of Medicine], <1987 - 02/20/2025>

(((SIV[Title] OR "simian immunodeficiency virus"[Title] OR SIV[MeSH Terms] OR HIV[Title] OR HIV-1[Title] OR "human immunodeficiency virus"[Title] OR AIDS[Title] OR "acquired immunodeficiency syndrome"[Title] OR HIV[MeSH Terms] OR "Acquired Immunodeficiency Syndrome"[MeSH Terms]) AND ("rhesus macaque"[Title/Abstract] OR "Macaca mulatta"[MeSH Terms] OR macaque[Title/Abstract]))) AND ("delta-9-tetrahydrocannabinol"[Title/Abstract] OR "Δ-9-tetrahydrocannabinol"[Title/Abstract] OR THC[Title/Abstract] OR "Delta-9-tetrahydrocannabinol"[MeSH Terms]) AND ((gene expression[Title/Abstract] OR mRNA[Title/Abstract] OR microRNA[Title/Abstract] OR miRNA[Title/Abstract] OR transcriptome[Title/Abstract] OR epigenetic*[Title/Abstract] OR methylation[Title/Abstract] OR histone*[Title/Abstract] OR transcription*[Title/Abstract] OR RNA-seq[Title/Abstract] OR chip-seq[Title/Abstract]) OR ("immune marker*"[Title/Abstract] OR "viral load"[Title/Abstract])))
