# Supplementary material for: Transcriptomic Alterations Induced by Tetrahydrocannabinol in SIV/HIV Infection: A Systematic Review
Source: Int J Mol Sci. 2025 Mar 13;26(6):2598. doi: 10.3390/ijms26062598 (PMC11942185; doi:10.3390/ijms26062598)
Supplement: Supplementary file 1 [file ijms-26-02598-s001.zip › Table S2.pdf]

Supplementary Table S2

The modified version of the Q-Genie tool

The modified version of the Q-Genie tool used for assessing the methodological and reporting quality of the included studies and the criteria for rating each item.

Item	Question and criteria for rating
**Q1**	Rate the study on the adequacy of the presented hypothesis and rationale.<br>**Best-case scenario:** The rationale, necessity, and hypotheses behind the study were adequately explained and were in line with previous findings and evidence.
**Q2**	Rate the study on the technical aspects of the measurement of the outcome.<br>**Best-case scenario:** If applicable, appropriate preprocessing methods were used. Validation methods were implemented for the most prominent findings. The methods were not likely to be biased. Technical aspects were reported appropriately.
**Q3**	Rate the study on the description of comparison groups.<br>**Best-case scenario:** If applicable, the age, sex, ethnicity, weight, and other underlying disorders of the subjects were clearly described.
**Q4**	Rate the study on the technical measures in sample recruitment to minimize confounding effects.<br>**Best-case scenario:** If applicable, the age, sex, ethnicity, weight, and other underlying disorders of the subjects were matched.
**Q5**	Rate the study on the technical measures to implement the intervention.<br>**Best-case scenario:** The dose and route of administration were reported and justified.
**Q6**	Rate the study on the disclosure and discussion of sources of bias.<br>**Best-case scenario:** Possible sources of bias and limitations in the sample recruitment and characteristics, intervention implementation, outcome measurement, and statistical analyses were discussed.
**Q7**	Rate the study on the description of planned analyses.<br>**Best-case scenario:** The analyses and tests performed in the study were described sufficiently and clearly.
**Q8**	Rate the study on the statistical methods.<br>**Best-case scenario:** If applicable, corrections for the false discovery rate (FDR) and tests for the normality of the distribution were performed and appropriate parametric or non-parametric tests were used.
**Q9**	Rate the study on the description and test of all assumptions and inferences.<br>**Best-case scenario:** All assumptions were based on concrete evidence. Inferences of test results were adequate, clear, and technically plausible.
**Q10**	Rate the study on whether the conclusions drawn by the authors were supported by the results and appropriate methods.<br>**Best-case scenario:** The conclusions drawn for each test and analysis were reached through appropriate methods and were supported by their results.
